# Novel Technique for Confirmation of the Day of Ovulation and Prediction of Ovulation in Subsequent Cycles Using a Skin-Worn Sensor in a Population With Ovulatory Dysfunction: A Side-by-Side Comparison With Existing Basal Body Temperature Algorithm and Vaginal Core Body Temperature Algorithm

**DOI:** 10.3389/fbioe.2022.807139

**Published:** 2022-03-04

**Authors:** Hurst B. S., Davies K., Milnes R. C., Knowles T. G., Pirrie A.

**Affiliations:** ^1^ Carolinas Medical Center, Department of Assisted Reproduction, Charlotte, NC, United States; ^2^ Independent Fertility Nurse Consultant and Coach, Castle Bytham, United Kingdom; ^3^ Fertility Focus Inc. (Now viO HealthTech Inc.), Old Saybrook, CT, United States; ^4^ Faculty of Health Sciences, University of Bristol, Bristol, United Kingdom; ^5^ Fertility Focus Limited (now viO HealthTech Limited), Basepoint Business Centre, Warwick, United Kingdom

**Keywords:** ovulation, skin temperature, core body temperature, basal body temperature, ovulatory dysfunction, fertile window, vaginal sensor, ovulation algorithm

## Abstract

**Objective:** Determine the accuracy of a novel technique for confirmation of the day of ovulation and prediction of ovulation in subsequent cycles for the purpose of conception using a skin-worn sensor in a population with ovulatory dysfunction.

**Methods:** A total of 80 participants recorded consecutive overnight temperatures using a skin-worn sensor at the same time as a commercially available vaginal sensor for a total of 205 reproductive cycles. The vaginal sensor and its associated algorithm were used to determine the day of ovulation, and the ovulation results obtained using the skin-worn sensor and its associated algorithm were assessed for comparative accuracy alongside a number of other statistical techniques, with a further assessment of the same skin-derived data by means of the “three over six” rule. A number of parameters were used to divide the data into separate comparative groups, and further secondary statistical analyses were performed.

**Results:** The skin-worn sensor and its associated algorithm (together labeled “SWS”) were 66% accurate for determining the day of ovulation (±1 day) or the absence of ovulation and 90% accurate for determining the fertile window (ovulation day ±3 days) in the total study population in comparison to the results obtained from the vaginal sensor and its associated algorithm (together labeled “VS”).

**Conclusion:** SWS is a useful tool for confirming the fertile window and absence of ovulation (anovulation) in a population with ovulatory dysfunction, both known and determined by means of the timing of ovulation. The body site where the skin-worn sensor was worn (arm or wrist) did not appear to affect the accuracy. Prior diagnosis of known causes of ovulatory dysfunction appeared to affect the accuracy to a lesser extent than those cycles grouped into late ovulation and “early and normal ovulation” groups. SWS is a potentially useful tool for predicting ovulation in subsequent cycles, with greater accuracy obtained for the “normal ovulation” group.

## 1 Introduction

### 1.1 Clinical and Scientific Background

There are three main goals in the determination of ovulation for the purposes of improving the chances of conception: 1) to confirm the presence or absence of ovulation (anovulation) in a reproductive cycle, 2) to confirm the day on which ovulation occurred, and 3) to use this ovulation day in one reproductive cycle to predict the date of ovulation and the “fertile window” for the subsequent cycle in order to improve the chances of natural conception or timing of intervention. The fertile window is the span of time during a reproductive cycle when conception can take place. Based on the 5-day lifespan of sperm in the female reproductive tract and the up to 48-h lifespan of the unfertilized oocyte (egg), the window is largely deemed to run from 3 to 5 days prior to the day of ovulation up to 1–2 days after ovulation (Colombo and Masarotto, 2000).

The relationship between a temperature rise and ovulation, and the use of a charted single oral temperature recording taken first thing upon waking to retrospectively determine the presence and timing of ovulation was reported in the literature over 100 years ago (van de Velde, 1904; Harvey and Crockete, 1932; Tompkins, 1944). This methodology is generally referred to as basal body temperature (BBT), as it seeks to establish the consistent lowest (basal) temperature in a 24-h period from which to determine a rise. Body temperature is actually at its most stable and lowest during nighttime sleep, and the waking oral temperature was considered the best proxy for that nighttime temperature when the method was developed. The “three over six (TOS) rule” originally proposed by Barrett and Marshall (Barrett and Marshall, 1969) and as further developed (McCarthy and Rockette, 1983) is the most widely used current method for determining the presence and timing of ovulation with the BBT technique. The basic rule holds that ovulation has occurred if in any window in the cycle there is a sustained rise in temperature over 3 consecutive days, which is at least 0.3°C (0.54°F) higher than the previous 6 consecutive days. The day of ovulation is determined to be the day prior to the first of the 3 “high” temperatures. This method is in effect a simple time and mathematical-based algorithm. Traditional clinical thinking holds that the temperature rise is associated with the thermogenic effect of released progesterone and that progesterone starts being released after a follicle has ruptured during ovulation and the corpus luteum starts to form. Following this logic, clinicians have long believed a temperature rise can only take place *after* ovulation has occurred. Recent research calls this association into question and instead suggests that there is a progesterone rise of approximately 0.5 ng/ml prior to ovulation (Dozortsev and Diamond, 2020). Clinical publications have generally dismissed the use of other temperature curve characteristics such as the “nadir” (the lowest point of the curve) or “dip” prior to the rise for confirmation or prediction of ovulation (McCarthy and Rockette, 1983; Barron and Fehring, 2005). These are important considerations when assessing algorithmic techniques, which might assist us in better understanding both when a temperature rise has occurred and also more importantly when the temperature rise *is* occurring *as a cycle progresses*. The limitations of the BBT method, in particular for confirming the absence of ovulation, the exact day of ovulation, and for predicting a subsequent fertile window, have been widely reported (Lenton et al., 1977; Martinez et al., 1992; Barron and Fehring, 2005; Mazerolle et al., 2011). The method is especially problematic for women with ovulatory dysfunction for two reasons. Their temperature curves are generally more erratic, increasing the difficulty of interpreting charts and hence confirming 1) the presence or absence of ovulation and 2) the day of ovulation. The difficulty in the use of the ovulation day for predicting ovulation and the fertile window for a subsequent cycle—goal 3)—is compounded by the irregularity of their ovulation timing and or cycles, which makes it considerably less likely that ovulation will re-occur on the same day in a subsequent cycle even if it could be established accurately in the first place (Ayres-de-Campos et al., 1995).

### 1.2 Research Topic

In recent years, temperature sensors have been developed, which allow the gathering of multiple temperature measurements over a period of time from either the skin or within the vagina. These sensors upload data to a mobile device app either automatically or by user-initiated transfer. They potentially eliminate some of the inaccuracy associated with the waking oral measurement used in the BBT method in three ways. Firstly, they can be “worn” overnight—when the body temperature is at its most stable and lowest level. Secondly, by using an industrial temperature measurement component (thermistor), they can, in theory, record at a higher temperature resolution (enabling the steps between each temperature value to be better understood), and at a higher accuracy than an oral thermometer. Thirdly, they can be “worn” throughout the night, enabling multiple readings to be taken—providing a better understanding of the most representative overnight temperature from which to calculate a temperature shift for each successive night. The sensors combined with the computing power of mobile devices make the application of algorithmic techniques much easier, both in determining the most representative overnight temperature and in understanding the temperature rise as the cycle progresses. Various publications have attempted to assess the accuracy of oral temperature (Freundl et al., 2003), skin temperature recorded on the top of the wrist (Goodale et al., 2019; Zhu et al., 2021), and vaginal temperature (Papaioannou et al., 2013a; Papaioannou et al., 2013b; Papaioannou et al., 2014; Regidor et al., 2018) for the determination of the date of ovulation by different statistical techniques, comparing the test method variously with ultrasound ovarian follicle measurements (to determine the date of ovulation by estimated date of follicle rupture), urinary luteinizing hormone (LH) results (to determine the date of ovulation as 24–48 h following two positive results), and BBT using the “TOS” rule. Although these comparator methods provide imperfect estimates of the date of ovulation, the results are nonetheless useful with a larger volume of cycles in determining the likelihood that the test method has provided a good understanding of the presence or absence of ovulation, and the day of ovulation. An estimate of percentage accuracy of each test method using data from the various statistical techniques employed by these publications has been made by the authors, by applying the principle of identifying true positives (TPs), true negatives (TNs), false positives (FPs), and false negatives (FNs) of the test method vs. the comparator method in the calculation (TP + TN)/(TP + TN + FP + FN). F score was also calculated to provide a comparison to studies where this was provided but the detailed results were not, using the calculation TP/TP + ½(FP + FN). The detailed results are recorded in Supplementary Table S1, and the details of the studies including comparator methods are recorded in Supplementary Table S2. The calculations based on these clinical papers as outlined in Supplementary Table S1 show that oral temperature is up to 78% accurate in determining the fertile window rather than the day of ovulation with an F score of 0.88, skin temperature recorded on the top of the wrist provides an F score of 0.78 in determining the fertile window, and vaginal temperature is up to 99% accurate in determining the actual day of ovulation, with an F score of 0.99. The greater accuracy of the vaginal temperature method and its ability to determine the day of ovulation rather than the less stringent fertile window is understood to be a result of it providing a better proxy for true core body temperature without the external influences and signal “noise” that affect oral and skin temperature (Baker et al., 2020) and that core body temperature is likely to provide a more accurate reflection of the temperature rise associated with the release of progesterone (Coyne et al., 2000). It should be noted that clinical studies show that the accuracy of all these temperature methods can be improved by the inclusion of a secondary method of ovulation confirmation, and the combination of temperature with cervical mucus observation is particularly relevant (Frank-Herrmann et al., 2007), although not the subject of this paper.

### 1.3 Objectives

We aimed to determine the accuracy of a novel algorithm combined with a newly developed skin-worn-sensor (together with the “SWS”: OvuFirst™—viO HealthTech Limited and Inc.). SWS determines the most representative overnight temperature, and then algorithmically confirms the presence or absence of ovulation and the date of ovulation. SWS was compared with the “TOS” rule for assessing the presence and timing of ovulation by eye on the same overnight representative data (“TOS”). SWS and TOS were then separately compared with an independent measurement of overnight vaginal temperature and its associated algorithm (“VS”: OvuSense™ OvuCore, viO HealthTech Limited and Inc.). In these comparative analyses VS was treated as the “gold standard”. SWS and VS were worn “side by side” by each participant with measurements taken each night of each recorded cycle except during menstruation and until ovulation was confirmed by both SWS and VS methods.

We aimed to compare the accuracy of the “Training Set” (an initial 93 cycles used to develop, test, and iterate the SWS algorithm) against the “Additional Set” (a further 112 cycles used for comparative testing after the Training Set was concluded). The methodology of these data sets shall be explained in the report.

We aimed to compare the accuracy of SWS for arm and wrist positioning modalities.

We aimed to separately compare SWS accuracy for four groups of reported prior diagnoses for the participants (Prior diagnosis of Polycystic Ovarian Syndrome (“PCOS”), “Hypothyroid,” “PCOS and Hypothyroid,” and “Confirmed No Diagnosis”), and the timing of ovulation.

We aimed to further examine the accuracy of TOS and SWS in predicting the date of ovulation in subsequent cycles compared with the actual VS confirmed day of ovulation for that subsequent cycle. By way of comparison and illustration of the potential difficulty in using prior cycle ovulation confirmation for prediction of ovulation in subsequent cycles, we aimed to examine the accuracy of VS in predicting the date of ovulation in subsequent cycles with the actual VS confirmed day of ovulation for those subsequent cycles.

## 2 Methods

### 2.1 Study Population

The volunteers for the study were recruited from the current userbase of the VS product in the United States and the United Kingdom at the time of recruitment, self-selected by responding to an advert in the VS product user group and answering an initial questionnaire. The volunteers consented to use the SWS alongside the VS to enable the collection of side-by-side results and as such were a convenience sample from within the users of the product. No financial incentive was provided to participate in the study.

The volunteers were randomly assigned a recording skin site of “arm” or “wrist” for SWS with the intention of ensuring approximately 25% of the study population were assigned to the “wrist” site and sent a “one size fits all” wristband along with their SWS sensor, with those assigned to the “arm” site being allocated one of three armband sizes according to their declared arm circumference: *Size D* 8 to <23 cm (7 to <9 in.); *Size F* 23 to <31 cm (9 to <12 in.); and *Size G* 31–38 cm (12–15 in.) and larger. Hence, there were four separate cohorts. Twenty volunteers received a wristband, 2 received armband *Size D*, 19 received armband *Size F*, and 46 received armband *Size G.* Note: although nominally each band size cohort (wrist, arm D, arm F, and arm G) should represent 25% of the total study population, it was clearly not possible to randomize the “arm” site group to the 3 armband sizes. Therefore, all those using an armband were treated as a single “skin site” group.

As part of the consent questionnaire, the volunteers were also asked to record existing prior diagnoses for “PCOS,” “Hypothyroid,” “PCOS and Hypothyroid,” and “Confirmed No Diagnosis.”

The 87 volunteers started the side-by-side recordings, of which 7 failed to eventually complete one registered full cycle of data (6 armband *Size F* and 1 armband *Size G*) and were excluded from the analysis. The remaining 80 volunteers recorded one or more cycles of side-by-side data. These 80 analysis participants recorded a total of 205 included cycles. They had an age range of 22–46 years at the start of the study, an average age of 32, and a median age of 32, with 95% CI 31.3–33.3. Fifty-three were from the United States, and 27 were from the United Kingdom.

As the study progressed, a “Training Set” of 93 cycles from 59 participants was used to develop, test, and iterate the SWS algorithm. Once the algorithm development was concluded, these data were set aside as a comparator for future recorded cycles. These 59 participants had an age range of 23–42 years at the start of the study, an average age of 33, and a median age of 33, with 95% CI 33.2–33.7. Thirty-nine Training Set participants were from the United States, and 20 were from the United Kingdom.

As users of the VS device, they were naturally biased towards a longer period of trying to conceive (TTC). For the 205 included cycles, the initial declared TTC time plus usage time for VS prior to the start of the study was calculated, with the demographic spread shown in Table 1.

**TABLE 1:** Study population cycle comparison by number of years trying to conceive.

	0–1 year	1–2 years	2–3 years	3–4 years	4–5 years	5–6 years	6–7 years	>7 years	Not actively trying	Total
Number of cycles	14	64	39	20	22	12	22	4	8	205
Average cycle length	35.5	37.0	35.3	35.7	36.4	33.2	49.0	99.8	47.1	39.1
Median cycle length	33.5	32.0	30.0	32.0	30.0	31.5	29.5	43.5	40.0	32.0
Std Dev cycle length	15.6	17.3	15.8	16.4	17.2	11.3	59.2	119.4	25.6	30.1
Upper CI 95%	43.7	41.2	40.2	42.9	43.6	39.5	73.8	216.7	64.9	43.2
Lower CI 95%	27.3	32.8	30.3	28.5	29.2	26.8	24.3	−17.2	29.4	34.9
CI	8.2	4.2	5.0	7.2	7.2	6.4	24.7	117.0	17.7	4.1

The average cycle lengths for the 6–7 years group were increased by two cycles of 167 and 279 days in length, and the average cycle length for the >7 years group was increased by one cycle of 290 days in length. Although these long cycles might be regarded as outliers and excluded from similar analyses in previous studies, the authors felt it was important to fully reflect the study population results including long negative (anovulatory) cycles with temperature fluctuations, which might create FPs in the comparator methods, and the authors decided to therefore retain them for this analysis.

A Kruskal–Wallis test was performed to determine if median cycle length was significantly different for the nine time TTC groups.

The test revealed that the median cycle length was not significantly different (H = 1.793, *p* = 0.877), and hence, these groups were thus treated as one in all subsequent analyses.

### 2.2 Materials

The VS and SWS sensors are proprietary devices using off-the-shelf industrial components. Both comply with Thermometer Standards ASTM E1112 and ISO 80601-2-56:2017+A1:2020.

Independent laboratory testing of the production VS device in simulated physiological conditions was conducted for the purposes of the manufacturer’s original FDA 510(k) submission in the United States and CE marking Technical File for certification as a class II medical device in Europe. This established a temperature measurement resolution (steps between each temperature value measured) of 0.003°C and an accuracy of 0.05°C for the VS. The SWS sensor uses an identical thermistor and circuitry to VS. SWS was tested in-house against VS under matching laboratory conditions to the original VS tests, and details were added to the regulatory Technical File for the product line in order to provide regulatory qualification for use in the study. A first production batch of the SWS sensor was produced.

The participants “wore” the SWS sensor under the armpit or on the underside of the wrist, and VS vaginally.

Data were downloaded from each sensor to a study version of the mobile device application each morning by the participants. Data were uploaded to an encrypted database, with a portal allowing the authors to assess the cycle results visually and with calculated results.

### 2.3 Algorithm Development

An initial “Training Set” of 93 cycles included in this study analysis was used to develop, test, and iterate the SWS algorithm. (Note: 2 additional cycles from 2 additional volunteers who produced positive results for VS were included for SWS development purposes originally but excluded from the study analysis due to having no confirmed cycle end date.)

The algorithm development examined three broad techniques with the representative overnight temperatures:

• a straight mathematical application of the “TOS” rule
• a moving average method that adds each new night’s data to a “window” as the cycle progresses and drops the oldest night, enabling an assessment of temperature rise by means of the change from one window to the next window
• a “spring-loaded beam” method*, which uses all the previous recordings in the cycle with the aim of lowering the overall impact of sample noise (for example, due to changes in sensor position at its particular skin site, sensor contact, alcohol consumption, and sleep times), but maximizing the sensitivity to general trends. *Note: this is the authors’ own nomenclature, which attempts to describe the way in which the algorithm works.

### 2.4 Data Analysis—Inclusion/Exclusion Criteria

Completed ovulatory and anovulatory cycles for which a cycle end date was logged by the participant with sufficient serial recordings for both sensors were included.

All cycles where a cycle end date was not logged by the participant were excluded.

### 2.5 Statistical Methods

Each cycle for each user was treated as an independent statistical unit as has been standard practice in clinical papers examining methods of determining ovulation in a study population.

Where confirmation of ovulation occurred by VS, the cycle was reported as a positive, and the VS reported date was deemed to be the date of ovulation. Where VS was unable to confirm ovulation, the cycle was reported as a negative (in other words anovulatory).

Two main methods were used for analyzing the resulting data and groups: a “Days Difference Method” and a “Threshold Method,” as follows.

#### 2.5.1 Days Difference Method

In order to provide an understanding of the statistical validity of the positive cycle results (those for which a day of ovulation was confirmed by VS) an analysis was conducted to show the difference between the day of ovulation as confirmed by TOS and SWS from the VS confirmed day of ovulation.

The analysis shows both the mean number of days difference (and therefore how far out the results are from VS) and also whether the results are biased towards earlier ovulation confirmation (negative days) or later ovulation confirmation (positive days) in the cycle for each of the TOS and SWS methods in comparison to VS.

Average Days difference (between TOS and SWS result compared with VS result) and the Standard Deviation of Days were calculated together with upper and lower 95% confidence bounds. An equal number of earlier and later days difference would of course produce a mean difference of zero; hence, it is important to assess the standard deviation alongside the mean days difference result to understand how far from the VS result each of the SW results was.

#### 2.5.2 Threshold Method

Sensitivity, specificity, accuracy, positive predictive value (PPV), negative predictive value (NPV), and an F score were reported for each of the TOS and SWS methods compared with VS results for 2 windows: TP result within ±1 day of the VS result and a TP result within ±3 days of VS result. The ±1 day result was deemed to be a valid TP equivalent result to VS (as ovulation can in reality occur at any point within a 24-h period and a result is only available once in every 24-h period), and the ±3 days result was deemed to be a valid TP for the fertile window (3 days either side of the date of ovulation as determined by VS).

Results other than TPs were classified as follows:

FP = a positive (+ve) result outside of these windows; or SWS and/or TOS +ve, VS negative (−ve)

TN = concordance between TOS and/or SWS and VS that no ovulation took place

FN = SWS and/or TOS −ve, VS +ve

The following formulae were then used for calculations:

Sensitivity = TP/(TP + TN)

Specificity = TN/(FP + TN)

PPV = TP/(TP + FN)

NPV = TN/(TN + FN)

These calculations can be interpreted as follows.

The sensitivity shows the percentage of positive ovulations for TOS or SWS, which correctly matches with the VS positive results within the ±1 day and ±3 days thresholds. The specificity shows the percentage of absent ovulations (anovulatory cycles) for TOS or SWS, which correctly match with the VS negative (anovulatory) results. The PPV results show the percentage of detected ovulations TOS or SWS get right compared with VS within the ±1 day and ±3 days thresholds. The NPV is the percentage of absent ovulations (anovulatory cycles) TOS or SWS detect correctly compared with VS.

However, the most important overall results in developing SWS are measures of accuracy. The accuracy figure shows how many positive and absent ovulations (anovulatory results) TOS or SWS match the VS results and within the ±1 day and ±3 days thresholds for the ovulatory cycles. It is a good measure of accuracy for a binary diagnostic test when TPs and TNs are deemed to be most important. The F score provides a useful alternative measure of accuracy, which is calculated using the precision and recall of the test. The precision is the same as the PPV, and the recall is the same as sensitivity. The F score is more appropriate for unbalanced populations where FPs and FNs are felt to be more important.

The following formulae were used for these calculations:

Accuracy = (TP + TN)/(TP + FP + TN + FN)

F score = TP/TP + ½(FP + FN).

The upper and lower 95% CIs were calculated for the accuracy and F score results to provide the likely range of values around the population mean.

### 2.6 Data Group Analyses

#### 2.6.1 Data Sets

From this initial data analysis carried out on the “Training Set,” the final version of the “spring-loaded beam” algorithm was chosen as the most accurate SWS method in comparison to VS and was fixed for the purposes of further testing. Participants were encouraged to continue side-by-side recordings using both the skin-worn and vaginal sensors, and some participants joined the study after the Training Set was frozen. The same analysis was then conducted for this “Additional Set” and then the “Combined Set” comprising all data to provide an overall assessment of the accuracy for the SWS as a marketable product.

The “Training Set” and “Additional Set” groups were tested for the significant difference by means of a Mann–Whitney test applied to the median cycle length.

The TOS and SWS methods themselves were then each tested for significant differences for their positive ovulation confirmation performance in comparison to VS between the “Training Set” and “Additional Set” groups. This was done by means of a Mann–Whitney test applied to the absolute Days Difference produced by the method outlined in Section 2.5.1 between the TOS and VS confirmed day of ovulation and the SWS and VS confirmed day of ovulation, in each case ignoring the negative sign, as appropriate.

#### 2.6.2 Skin Site Groups

A further analysis for “Arm” and “Wrist” groups was conducted. This analysis is labeled Skin Site. The Arm and Wrist groups were tested for the significant difference by means of a Mann–Whitney test applied to the median cycle length.

The TOS and SWS methods themselves were then each tested for significant differences for their positive ovulation confirmation performance in comparison to VS between the “Arm” and “Wrist” groups. This was done by means of a Mann–Whitney test applied to the absolute Days Difference produced by the method outlined in Section 2.5.1 between the TOS and VS confirmed day of ovulation and the SWS and VS confirmed day of ovulation, in each case ignoring the negative sign, as appropriate.

#### 2.6.3 Diagnosis and Ovulation Timing Groups

Seventy participants volunteered to provide information on their prior diagnoses at the start of the study and were grouped accordingly into the categories: “PCOS,” “Hypothyroid,” “PCOS and Hypothyroid,” and “Confirmed No Diagnosis” diagnostic results. For the 172 included cycles from these 70 participants, the median cycle values were assessed as shown in Table 2.

**TABLE 2:** Cycle comparison by prior diagnosis group.

	PCOS	Hypothyroid	PCOS + Hypothyroid	Confirmed No Diagnosis
Number of cycles	106	7	6	53
Average cycle length	40.9	31.3	76.2	34.5
Median cycle length	32.0	32.0	35.0	29.0
Std Dev cycle length	30.1	4.0	104.8	16.6
Upper CI 95%	46.6	34.2	160.0	39.0
Lower CI 95%	35.2	28.3	−7.7	30.1

Note. PCOS, polycystic ovarian syndrome.

A Kruskal–Wallis test revealed that the median cycle length was significantly different (H = 7.676, *p* = 0.053) between the four groups.

However, forty-three participants reported a prior diagnosis of PCOS, but only 2 reported hypothyroid diagnosis, and 4 reported both diagnoses. These smaller numbers produced anomalous results (e.g., both produced 100% accuracy for SWS in the ±3 days Threshold Analysis). As further described in the *Results* section, these four groups were therefore amalgamated into “Confirmed Prior Diagnosis” and “Confirmed No Diagnosis” groups and tested for the significant difference by means of a Mann–Whitney test applied to the median cycle length.

A further analysis was performed for VS confirmed positive results using the criteria of “Late Ovulation” as defined by all cycles, where the VS ovulation date was greater than 65% of the cycle length*. An analysis of “Early Ovulation” was also made using a <40% threshold, but only 6 cycles showed this characteristic. These cycles were therefore aggregated with those with ovulation confirmed between 40% and 65% of the cycle length in *Early + Normal Ovulation Timing* group for comparative purposes. This analysis is labeled “Diagnosis and Ovulation Timing.” The “Early + Normal Ovulation Timing” and “Late Ovulation” groups were tested for the significant difference by means of a Mann–Whitney test applied to the median cycle length. *For example, day 20 ovulation in a 30-day cycle is 20/30 = 66.7% of the way through the cycle and therefore labeled as “late” for this analysis.

The TOS and SWS methods themselves were then each tested for significant difference for their positive ovulation confirmation performance in comparison to VS between each pair of groups: “Confirmed Prior Diagnosis” and “Confirmed No Diagnosis,” and “Early + Normal Ovulation Timing” and “Late Ovulation.” This was done by means of a Mann–Whitney test applied to the absolute Days Difference produced by the method outlined in Section 2.5.1 between the TOS and VS confirmed day of ovulation and the SWS and VS confirmed day of ovulation (ignoring the negative sign, as appropriate).

#### 2.6.4 Ovulation Prediction

Lastly, the analysis for the prediction of ovulation in a subsequent cycle was conducted. Only positive results are included (as predicting a negative result has no meaning in this context); hence, accuracy and specificity can be calculated by omitting TNs, along with PPV and F score using the calculations above.

The subsequent cycle ovulation prediction method has been adapted by VS and SWS algorithms and was applied manually to the data for TOS for the purposes of this study. The prediction method works as follows: when a user enters a new cycle start date, the median day of ovulation confirmation from the up to 12 previous ovulatory cycles (those for which an ovulation confirmation occurred) is used to predict the ovulation day for the cycle that has just started, with cycles containing no ovulation confirmation ignored. As the data are not continuous, the algorithm never picks a median ovulation day value that has not previously actually occurred, so in the case of an even number of previous ovulatory cycles, the higher of the two values which sit in the middle of the array is taken. This analysis is labeled “Subsequent Cycle Prediction.”

As confirmation and prediction are separate algorithmic methods, the authors felt it was important that in order to judge how accurately TOS and SWS methods predict ovulation as confirmed by the VS result for a subsequent cycle, VS was also examined for its own ability to predict ovulation compared with its separate confirmed ovulation date for the subsequent cycle. Both TOS and SWS prediction compared with VS subsequent cycle confirmation and VS prediction compared with VS subsequent cycle confirmation are therefore included.

The TOS and SWS methods themselves were each tested for significant differences for their positive ovulation prediction performance in comparison to VS between each pair of groups: “Confirmed Prior Diagnosis” and “Confirmed No Diagnosis,” and “Early + Normal Ovulation Timing” and “Late Ovulation.” This was done by means of a Mann–Whitney test applied to the absolute Days Difference produced by the method outlined in Section 2.5.1 between the TOS and VS predicted day of ovulation and the SWS and VS predicted day of ovulation (ignoring the negative sign, as appropriate).

## 3 Results

### 3.1 Data Sets

As outlined in Table 3, median cycle lengths in the Training Set and Additional Set groups were 32 and 31 days, respectively. However, the distributions in the two groups for which cycle lengths could be determined differed significantly (Mann–Whitney U = 4,285.5, n1 = 93, n2 = 112, *p* = 0.0291), and therefore, for the purpose of this analysis, this may account for a difference in results between the two groups.

**TABLE 3:** Cycle comparison by data set groups.

	Combined set	Training set	Additional set
Total number of cycles	205	93	112
Average cycle length	39.1	44.8	34.3
Median cycle length	32.0	32.0	31.0
Std Dev cycle length	30.1	40.4	16.2
Upper CI 95%	43.2	53.0	37.3
Lower CI 95%	34.9	36.5	31.3
CI	4.1	8.2	3.0

Table 4 presents the comparison of absolute days difference between the TOS and SWS methods compared with VS for the Training, Additional, and Combined Data Sets. Participants were able to belong to both of these groups, as the Additional Set represented a follow-on data gathering. The mean days difference for both methods is a negative number indicating that, on average, both TOS and SWS methods are confirming ovulation earlier in the cycle than VS. The Training Set was intended to provide optimal results against the VS method, which would then allow SWS to perform consistently in a larger data set as the SWS product use increases. Notwithstanding the statistical difference in cycle lengths shown by the Mann–Whitney test, it is clear from the standard deviation results that there is a wider spread in the day of ovulation results obtained by TOS and SWS compared with VS in the Additional Set than the Training Set, although the mean difference is actually closer to the SWS result in the Additional Set for both methods.

**TABLE 4:** Days difference between TOS and SWS confirmed day of ovulation for ovulatory cycles compared with VS positive cycles for data sets.

	Combined set		Training set		Additional set	
+ve VS cycles in each group	158		75		83	
Participants with +ve VS cycles in each group	70		51		43	
*Method*	TOS	SWS	TOS	SWS	TOS	SWS
Mean days difference compared with VS	−3.26	−1.51	−4.05	−1.29	−2.52	−1.71
Standard deviation days compared with VS	1.93	1.83	1.31	1.05	1.44	1.51
Upper CI 95%	−2.96	−1.23	−3.76	−1.06	−2.21	−1.39
Lower CI 95%	−3.56	−1.80	−4.35	−1.53	−2.83	−2.04

Note. A single participant was able to take part in both the Training Set and the Additional Set; hence, each participant was able to have one or more cycles, which contributed to both groups, and the total number of participants added together for those two groups in the table above is therefore more than the 70 total participants with positive cycles.

Table 5 presents the Threshold Method analysis for the Training Set and Additional Set data, with the Combined Set of both data presented to provide complete results for the ±1 day and ±3 days thresholds compared with VS results.

**TABLE 5:** Threshold method results for TOS and SWS compared with VS for data sets.

	Combined set				Training set				Additional set			
+ve VS cycles	158				75				83			
−ve VS cycles	47				18				29			
Total cycles	205				93				112			
Participants	80				60				55			
*Thresholds*	±1 day		±3 days		±1 day		±3 days		±1 day		±3 days	
*Method*	TOS	SWS	TOS	SWS	TOS	SWS	TOS	SWS	TOS	SWS	TOS	SWS
TP	86	88	119	140	43	47	55	71	43	41	64	69
FP	53	63	20	11	23	26	11	2	30	37	9	9
TN	43	45	43	45	16	18	16	18	27	27	27	27
FN	23	9	23	9	11	2	11	2	12	7	12	7
Total cycles	205	205	205	205	93	93	93	93	112	112	112	112
Sensitivity	79%	91%	84%	94%	80%	96%	83%	97%	78%	85%	84%	91%
Specificity	45%	42%	68%	80%	41%	41%	59%	90%	47%	42%	75%	75%
PPV	62%	58%	86%	93%	65%	64%	83%	97%	59%	53%	88%	88%
NPV	65%	83%	65%	83%	59%	90%	59%	90%	69%	79%	69%	79%
Accuracy	62.9%	64.9%	79.0%	90.2%	63.4%	69.9%	76.3%	95.7%	62.5%	60.7%	81.3%	85.7%
Upper CI 95%	69.6%	71.4%	84.4%	93.9%	73.2%	79.0%	84.5%	98.8%	71.5%	69.8%	88.0%	91.6%
Lower CI 95%	55.9%	57.9%	72.8%	85.3%	52.8%	59.5%	66.4%	89.4%	52.9%	51.0%	72.8%	77.8%
F score	0.69	0.71	0.85	0.93	0.72	0.77	0.83	0.97	0.67	0.65	0.86	0.90
Upper CI 95%	0.77	0.79	0.90	0.97	0.83	0.87	0.91	1.00	0.78	0.77	0.93	0.95
Lower CI 95%	0.60	0.62	0.78	0.88	0.59	0.65	0.72	0.90	0.54	0.52	0.76	0.81

Note. A single participant was able to take part in both the Training Set and the Additional Set; hence, each participant was able to have one or more cycles, which contributed to both groups, and the total number of participants added together for those two groups in the table above is therefore more than the 80 total participants with positive and negative cycles.

TP, true positive; FP, false positive; TN, true negative; FN, false negative; PPV, positive predictive value; NPV, negative predictive value.

The ovulation confirmation performance of Days Difference distributions in the Training Set and Additional Set groups did not differ significantly for either TOS (*p* = 0.1948) or SWS (*p* = 0.3132), and therefore, despite the significant difference in median cycle lengths between the two groups, it is valid to compare the Days Difference and Threshold results for TOS and SWS between the two groups.

With an accuracy of 65%, SWS does not appear to be capable of accurate confirmation of the exact day of ovulation (when compared with the VS method), although it is shown by these data to be more sensitive than TOS (91% vs. 79%) for that ±1 day threshold. It should be noted that the accuracy of the TOS method is higher than that of the SWS for the ±1 day threshold but lower than that of the SWS for the ±3 days threshold. SWS has an accuracy of 90% and an F score of 0.93 for the Combined Group for the ±3 days threshold. The accuracy drops for ±3 days for the SWS method in the Additional Set. Given these populations are probably unbalanced, the F score may provide a better indicator of the TOS and SWS accuracy between the groups.

### 3.2 Skin Site

As outlined in Table 6, median cycle lengths in the Arm and Wrist groups were each 32 days. The distributions in the two groups did not differ significantly (Mann–Whitney U = 3,829.5, n1 = 153, n2 = 51, *p* = 0.7906). Median cycle lengths are therefore unlikely to account for any differences in results between the two groups.

**TABLE 6:** Cycle comparison by skin site groups.

	Arm	Wrist
Number of cycles	154	51
Average cycle length	39.0	39.3
Median cycle length	32.0	32.0
Std Dev cycle length	28.0	36.1
Upper CI 95%	43.4	49.2
Lower CI 95%	34.5	29.4

Table 7 presents the comparison of absolute days difference between the TOS and SWS methods compared with VS for the Arm and Wrist Modalities. Each participant was only able to belong to one of these distinct groups. The TOS method appears to have a large negative mean days difference compared with VS for the Arm Site in comparison to the SWS results for both Skin Site groups and the TOS Method for the Wrist Site Group.

**TABLE 7:** Days difference between TOS and SWS confirmed day of ovulation for ovulatory cycles compared with VS positive cycles for skin site.

	Arm		Wrist	
+ve VS cycles in each group	118		40	
Participants with +ve VS cycles in each group	51		20	
*Method*	TOS	SWS	TOS	SWS
Mean days difference compared with VS	−3.78	−1.65	−1.69	−1.10
Standard deviation days compared with VS	1.70	1.66	0.94	0.79
Upper CI 95%	−3.47	−1.35	−1.40	−0.85
Lower CI 95%	−4.09	−1.95	−1.98	−1.35

Table 8 presents the Threshold Method analysis for the Arm and Wrist Skin Site groups.

**TABLE 8:** Threshold method results for TOS and SWS compared with VS for skin site.

	Arm				Wrist			
+ve VS cycles	118				40			
−ve VS cycles	36				11			
Total cycles	154				51			
Participants	60				20			
*Thresholds*	±1 day		±3 days		±1 day		±3 days	
*Method*	TOS	SWS	TOS	SWS	TOS	SWS	TOS	SWS
TP	61	61	87	103	25	27	32	37
FP	41	52	15	10	12	11	5	1
TN	33	34	33	34	10	11	10	11
FN	19	7	19	7	4	2	4	2
Total cycles	154	154	154	154	51	51	51	51
Sensitivity	76%	90%	82%	94%	86%	93%	89%	95%
Specificity	45%	40%	69%	77%	45%	50%	67%	92%
PPV	60%	54%	85%	91%	68%	71%	86%	97%
NPV	63%	83%	63%	83%	71%	85%	71%	85%
Accuracy	61.0%	61.7%	77.9%	89.0%	68.6%	74.5%	82.4%	94.1%
Upper CI 95%	68.8%	69.4%	84.2%	93.4%	80.9%	85.7%	91.6%	98.8%
Lower CI 95%	52.9%	53.5%	70.5%	82.9%	54.1%	60.4%	69.1%	83.8%
F score	0.67	0.67	0.84	0.92	0.76	0.81	0.88	0.96
Upper CI 95%	0.77	0.77	0.90	0.97	0.89	0.92	0.96	1.00
Lower CI 95%	0.56	0.57	0.75	0.86	0.58	0.63	0.73	0.84

Note. TP, true positive; FP, false positive; TN, true negative; FN, false negative; PPV, positive predictive value; NPV, negative predictive value.

The ovulation confirmation performance of Days Difference distributions in the Arm and Wrist groups did not differ significantly for either TOS (*p* = 0.2647) or SWS (*p* = 0.0953), and therefore Mann–Whitney tests on both median cycle length and absolute days difference would indicate that it is valid to compare the Days Difference and Threshold results for TOS and SWS between the two groups.

As with the Training and Additional Data Set results, SWS produces a higher accuracy for both groups than TOS. Given the lack of statistical significance between the two groups, it would appear that the Wrist Site provides a higher accuracy for SWS than the Arm Site in terms of both the accuracy and F score results (±3 days threshold accuracy 94% and F score 0.96 Wrist, accuracy 89% and F score 0.92 Arm).

### 3.3 Diagnosis and Ovulation Timing

Eight participants accounting for 33 cycles provided no data on prior Diagnosis and were excluded from the Diagnosis analysis. Forty-seven anovulatory cycles were excluded from the Ovulation Timing analysis.

A total of 22 participants with Confirmed Prior Diagnosis recorded one or more ovulatory cycles with Late Ovulation, and a further 11 recorded only anovulatory cycles. A further 10 participants with Confirmed No Diagnosis or who did not provide an answer on prior diagnosis recorded one or more cycles with Late Ovulation, and a final additional 2 participants with Confirmed No Diagnosis or did not provide an answer on prior diagnosis recorded only anovulatory cycles. These total 45 participants could each be regarded as having potential ovulatory dysfunction. However, 17 participants with Confirmed Prior Diagnosis recorded only Early + Normal Ovulation Cycles, so although these participants might be expected to exhibit signs of ovulatory dysfunction, they were not shown to do so in this study. These participants accounted for 100 cycles with signs of ovulatory dysfunction (53 Late Ovulation and 47 anovulatory cycles), and a further 54 cycles with Short + Normal Ovulation Timing recorded by participants indicating a Confirmed Prior Diagnosis. With over half the 80 participants and just under half the 205 cycles in the study showing signs of ovulatory dysfunction, the population can be expected to record less accurate results than in a population with no diagnosis, or normal ovulation timing, i.e., those without expected ovulatory dysfunction. These analyses in this section seek to examine the differences between these groups.

**FIGURE 1:**
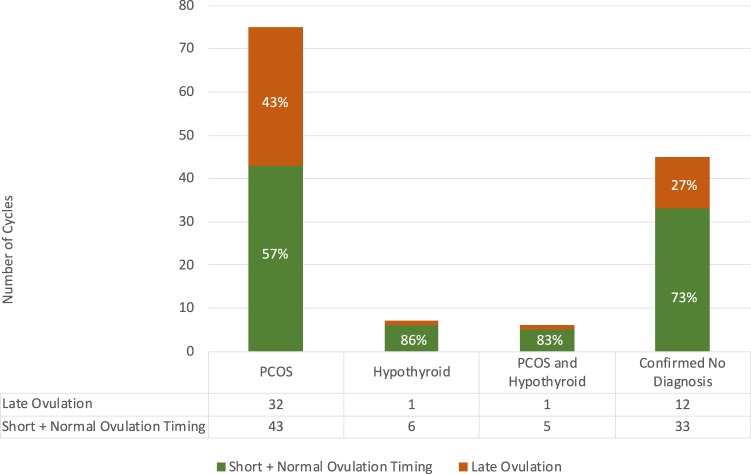
Relationship between ovulation timing and confirmed diagnosis in the study population with numbers of cycles per category tabulated.

The association between Diagnosis and Ovulation Timing cycles is shown in Figure 1, demonstrating that although Late Ovulation was more prevalent for 43% of those who declared a prior diagnosis of PCOS, it still occurred in over ¼ cycles for those who declared no prior diagnosis associated with ovulatory dysfunction. No comment can be made on the ovulation timing splits for the Hypothyroid and PCOS and Hypothyroid groups due to the small sample sizes.

Table 9 presents the cycle comparison for the Diagnosis and Ovulation Timing Groups.

**TABLE 9:** Cycle comparison by diagnosis and ovulation timing groups.

	Confirmed prior diagnosis	Confirmed No diagnosis	Late ovulation	Early + normal ovulation timing
Number of cycles	119	53	53	105
Average cycle length	42.1	34.5	38.5	34.4
Median cycle length	33.0	29.0	36.0	30.0
Std Dev cycle length	34.5	17.1	18.3	25.6
Upper CI 95%	48.3	39.1	43.4	39.3
Lower CI 95%	35.9	29.9	33.6	29.5
CI	6.2	4.6	4.9	4.9

Median cycle lengths in the Confirmed Prior Diagnosis and Confirmed No Diagnosis groups were 33 and 29 days, respectively. The distributions in the two groups differed significantly (Mann–Whitney U = 2,405.5, n1 = 119, n2 = 53, *p* = 0.0131), and therefore for the purpose of this analysis, the differences in results between the two groups may be a result of that statistical difference in median cycle lengths for the group populations.

Median cycle lengths in the Late Ovulation and Early + Normal Ovulation Timing groups were 36 and 30 days, respectively. The distributions in the two groups for which cycle lengths could be determined differed highly significantly (Mann–Whitney U = 1,470.5, n1 = 53, n2 = 105, *p* < 0.001), and therefore for the purpose of this analysis, the differences in results between the two groups may be a result of that statistical difference in median cycle lengths for the group populations.

Table 10 presents the comparison of absolute days difference between the TOS and SWS methods compared with VS for those participants for the Diagnosis and Ovulation Timing groups.

**TABLE 10:** Days difference between TOS and SWS confirmed day of ovulation for ovulatory cycles compared with VS positive cycles for diagnosis and ovulation timing groups.

	Confirmed prior diagnosis		Confirmed No diagnosis		Late ovulation		Early + normal ovulation timing	
+ve VS cycles in each group	88		45		53		105	
Participants with +VS cycles in each group	40		21		37		53	
*Method*	TOS	SWS	TOS	SWS	TOS	SWS	TOS	SWS
Mean days difference compared with VS	−2.98	−1.24	−3.45	−2.93	−7.09	−3.26	−1.28	−0.63
Standard deviation days compared with VS	1.35	1.45	0.98	0.73	0.84	1.02	1.75	1.53
Upper CI 95%	−2.69	−0.93	−3.17	−2.72	−6.87	−2.99	−0.95	−0.34
Lower CI 95%	−3.26	−1.54	−3.74	−3.15	−7.32	−3.54	−1.62	−0.92

Each participant was only able to belong to one of the Confirmed Prior Diagnosis (by answering the study survey indicating PCOS, PCOS, and Hypothyroid, or Hypothyroid) or Confirmed No Diagnosis group. The spread of results as indicated by the standard deviation of days appears to be larger in the Confirmed Prior Diagnosis group. Despite the mean days difference being higher in the Confirmed No Diagnosis group, there appears to be a closer overall agreement between TOS and SWS with VS as indicated by the lower standard deviation of days.

For absolute days difference between the TOS and SWS methods compared with VS for the Late Ovulation and Early + Normal Ovulation Timing groups, each participant was able in theory to contribute cycles to both groups, so there is a degree of crossover between the population despite exhibiting different cycle characteristics in order to contribute to one or other group. The spread of results as indicated by the standard deviation of days appears to be lower in the Late Ovulation group, whereas the negative mean days difference is higher in this group, indicating closer overall agreement between TOS and SWS with VS for the Late Ovulation group but a larger overall shift to earlier ovulation confirmation by TOS and SWS.

Table 11 presents the Threshold Method analysis for the Diagnosis and Ovulation Timing groups.

**TABLE 11:** Threshold method results for TOS and SWS compared with VS for diagnosis and ovulation timing groups.

	Confirmed prior diagnosis				Confirmed No diagnosis				Late ovulation				Early + normal ovulation timing			
+ve VS cycles	88				45				53				105			
−ve VS cycles	31				8				0				0			
Total cycles	119				53				53				105			
Participants	50				22				37				53			
*Thresholds*	±1 day		±3 days		±1 day		±3 days		±1 day		±3 days		±1 day		±3 days	
*Method*	TOS	SWS	TOS	SWS	TOS	SWS	TOS	SWS	TOS	SWS	TOS	SWS	TOS	SWS	TOS	SWS
TP	49	48	64	76	24	27	36	41	28	31	39	46	58	57	80	94
FP	27	35	12	7	15	15	3	1	14	18	3	3	35	43	13	6
TN	28	30	28	30	8	8	8	8								
FN	15	6	15	6	6	3	6	3	11	4	11	4	12	5	12	5
Total cycles	119	119	119	119	53	53	53	53	53	53	53	53	105	105	105	105
Sensitivity	77%	89%	81%	93%	80%	90%	86%	93%	72%	89%	78%	92%	83%	92%	87%	95%
Specificity	51%	46%	70%	81%	35%	35%	73%	89%								
PPV	64%	58%	84%	92%	62%	64%	92%	98%	67%	63%	93%	94%	62%	57%	86%	94%
NPV	65%	83%	65%	83%	57%	73%	57%	73%								
Accuracy	64.7%	65.5%	77.3%	89.1%	60.4%	66.0%	83.0%	92.5%	52.8%	58.5%	73.6%	86.8%	55.2%	54.3%	76.2%	89.5%
Upper CI 95%	73.2%	74.0%	84.5%	94.1%	73.5%	78.5%	91.9%	97.9%	66.7%	71.9%	84.7%	94.5%	65.0%	64.0%	84.0%	94.7%
Lower CI 95%	55.4%	56.3%	68.7%	82.0%	46.0%	51.7%	70.2%	81.8%	38.6%	44.1%	59.7%	74.7%	45.2%	44.3%	66.9%	82.0%
F score	0.70	0.70	0.83	0.92	0.70	0.75	0.89	0.95	0.69	0.74	0.85	0.93	0.71	0.70	0.86	0.94
Upper CI 95%	0.80	0.81	0.90	0.97	0.84	0.88	0.97	0.99	0.83	0.86	0.94	0.98	0.81	0.80	0.93	0.98
Lower CI 95%	0.58	0.58	0.72	0.84	0.52	0.58	0.75	0.84	0.53	0.58	0.71	0.82	0.60	0.59	0.78	0.88

Note. TP, true positive; FP, false positive; TN, true negative; FN, false negative; PPV, positive predictive value; NPV, negative predictive value.

The ovulation confirmation performance of Days Difference distributions in the Confirmed Prior Diagnosis and Confirmed No Diagnosis groups did not differ significantly for either TOS (*p* = 0.6518) or SWS (*p* = 0.3182), and therefore, despite the significant difference in median cycle lengths between the two groups, it is valid to compare the Days Difference and Threshold results for TOS and SWS between the two groups.

The ovulation confirmation performance of Days Difference distributions in the Late Ovulation and Confirmed Early + Normal Ovulation Timing groups did not differ significantly for either TOS (*p* = 1.9551) or SWS (*p* = 0.3601), and therefore, despite the significant difference in median cycle lengths between the two groups, it is valid to compare the Days Difference and Threshold results for TOS and SWS between the two groups.

The relative number of TN results in the Confirmed Prior Diagnosis group (24% of all cycles for TOS, 25% of all cycles for SWS) has resulted in a similar accuracy for the ±1 day threshold for this group (65% TOS, 66% SWS) compared with the Confirmed No Diagnosis group (60% TOS, 66% SWS) despite the difference in Standard Deviation days (SD) results for positive ovulations between the two groups as shown in Table 10. The relative number of FN results in the Confirmed No Diagnosis group (11% of all cycles for TOS and 6% of all cycles for SWS) accounts for the difference in the accuracy between the two methods for ±1 day threshold in the Confirmed No Diagnosis group. The accuracy for both groups is noticeably higher for SWS for the ±3 days Threshold.

No TN results are presented for the Ovulation Timing group analysis, as an ovulation confirmation has to take place in order to determine ovulation timing. FNs are still possible where the comparator method (TOS or SWS) provides a Negative result when VS showed a Positive result.

The relative number of FN results in the Late Ovulation group (21% of all cycles for TOS and 8% of all cycles for SWS) has created a disparity in the accuracy for the ±1 day threshold between TOS and SWS (53% TOS and 59% SWS) in comparison with the Standard Deviation days (SD) results for positive ovulations only of 0.84 TOS and 1.02 SWS. On the other hand, the same relative total FP and FN results in the Early and Normal Ovulation group (45% for both methods) have results in a similar accuracy with 55% TOS and 54% SWS for the ±1 day threshold vs. SD 1.75 TOS and 1.53 SWS. The accuracy for both groups is markedly higher for SWS for the ±3 days Threshold.

### 3.4 Subsequent Cycle Prediction

Thirty-seven participants had 85 serial positive cycles, which could be included in the prediction analysis.

**FIGURE 2:**
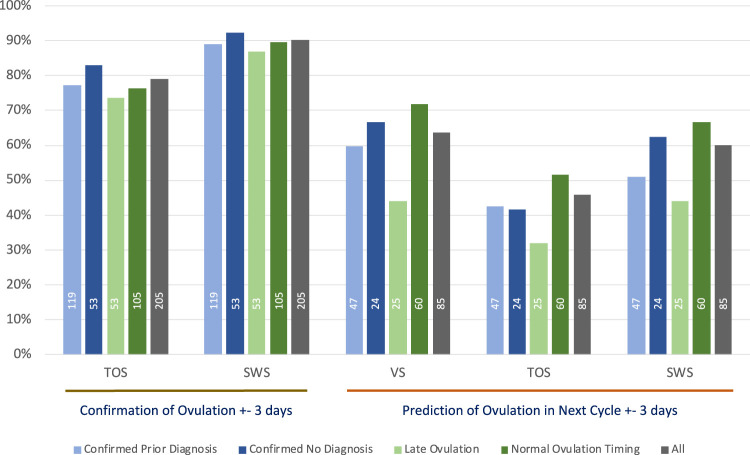
The ±3 days % accuracy of confirmation of ovulation and prediction of ovulation in subsequent cycles using median day of ovulation prediction method, annotated with numbers of cycles contributing to each analysis.

The percentage accuracy for predicting ovulation in a subsequent cycle within ±3 days is shown in Figure 2. The figure repeats the confirmation accuracy as outlined earlier in Section 3 and then shows the ability of each method to predict ovulation in each subsequent cycle with the algorithm technique detailed in Section 2.6.4. As VS is used as the gold standard in this study for confirmation, it is important to understand how well it is capable of predicting ovulation on the basis of its own data in prior cycles; hence, the prediction accuracy of VS compared with the separate VS subsequent cycle ovulation confirmation is included.

As outlined in Section 3.3, at least half the population in this study can be regarded as having a degree of ovulatory dysfunction. As for the expected lower accuracy with confirmation of ovulation, it would likewise be expected to produce lower accuracy for predicting ovulation by the method outlined in Section 2.6.4. The analyses in this section seek to further examine the groups that have signs of ovulatory dysfunction against those with no signs of ovulatory dysfunction.

The total number of cycles that contributed to each of the VS, TOS, and SWS methods for each group summarized in Figure 2 is shown as a legend within each relevant bar.

Table 12 outlines the results for the days difference in the prediction of ovulation using VS (against itself), TOS, and SWS previous cycle data for data in the study when compared to the VS result for a subsequent cycle. The analysis includes all data, and then the data are separated for Diagnosis and Ovulation Timing groups.

**TABLE 12:** Days difference between VS confirmed day of ovulation for ovulatory cycles compared with predicted day of ovulation by VS, TOS, and SWS using median day of ovulation prediction method for all data, and diagnosis and ovulation timing groups.

	All data			Confirmed prior diagnosis			Confirmed No diagnosis			Late ovulation			Early + normal ovulation timing		
+ve subsequent VS Cycles	85			47			24			25			60		
Participants with +ve subsequent cycles in each group	37			19			13			19			28		
*Method*	VS	TOS	SWS	VS	TOS	SWS	VS	TOS	SWS	VS	TOS	SWS	VS	TOS	SWS
Mean days difference compared with VS	−0.34	−1.74	−0.66	−0.34	−2.20	−0.94	−1.25	−1.85	−0.96	−4.80	−5.50	−4.88	1.52	−0.15	1.10
Standard deviation days compared with VS	7.22	7.20	7.43	9.00	9.03	9.27	4.46	4.35	4.76	11.06	11.07	11.36	3.52	4.15	3.92
Upper CI 95%	1.19	−0.21	0.92	2.23	0.38	1.71	0.53	−0.11	0.95	−0.46	−1.16	−0.43	2.41	0.90	2.09
Lower CI 95%	−1.88	−3.27	−2.24	−2.91	−4.78	−3.59	−3.03	−3.59	−2.86	−9.14	−9.84	−9.33	0.62	−1.20	0.11

The VS, TOS, and SWS methods for all data together have a high standard deviation for prediction, which is the result of very different actual ovulation results in subsequent cycles.

There is a noticeably higher days standard deviation for the Confirmed Prior Diagnosis group than the Confirmed No Diagnosis group.

There is a noticeably high standard deviation for the Late Ovulation group for all three methods.

Table 13 presents the Threshold Method analysis for the ovulation prediction based on previous cycles for all data, and Diagnosis and Ovulation Timing groups.

**TABLE 13:** Threshold method results for VS, TOS, and SWS ovulation prediction compared with VS subsequent cycle results using median day of ovulation prediction method for all data, and diagnosis and ovulation timing groups.

	All data			Confirmed prior diagnosis			Confirmed No diagnosis			Late ovulation			Early + normal ovulation timing		
+ve subsequent VS cycles	85			47			24			25			60		
Participants with +ve subsequent cycles in each group	37			19			13			19			28		
*Threshold*	±3 days			±3 days			±3 days			±3 days			±3 days		
*Method*	VS	TOS	SWS	VS	TOS	SWS	VS	TOS	SWS	VS	TOS	SWS	VS	TOS	SWS
TP	54	39	51	28	20	24	16	10	15	11	8	11	43	31	40
FP	31	35	34	19	20	23	8	10	9	14	14	14	17	21	20
TN	0	0	0	0	0	0	0	0	0	0	0	0	0	0	0
FN	0	11	0	0	7	0	0	4	0	0	3	0	0	8	0
Total cycles	85	85	85	47	47	47	24	24	24	25	25	25	60	60	60
Sensitivity	100%	78%	100%	100%	74%	100%	100%	71%	100%	100%	73%	100%	100%	79%	100%
PPV	64%	53%	60%	60%	50%	51%	67%	50%	63%	44%	36%	44%	72%	60%	67%
Accuracy	63.5%	45.9%	60.0%	59.6%	42.6%	51.1%	66.7%	41.7%	62.5%	44.0%	32.0%	44.0%	71.7%	51.7%	66.7%
Upper CI 95%	73.7%	57.0%	70.5%	73.6%	57.8%	65.9%	84.4%	63.4%	81.2%	65.1%	53.5%	65.1%	82.5%	64.8%	78.3%
Lower CI 95%	52.4%	35.0%	48.8%	44.3%	28.3%	36.1%	44.7%	22.1%	40.6%	24.4%	14.9%	24.4%	58.6%	38.4%	53.3%
F score	0.78	0.63	0.75	0.75	0.60	0.68	0.80	0.59	0.77	0.61	0.48	0.61	0.83	0.68	0.80
Upper CI 95%	0.87	0.75	0.85	0.87	0.76	0.82	0.94	0.82	0.93	0.83	0.74	0.83	0.92	0.81	0.90
Lower CI 95%	0.66	0.50	0.63	0.58	0.41	0.50	0.56	0.33	0.53	0.36	0.24	0.36	0.71	0.53	0.66

Note. TP, true positive; FP, false positive; TN, true negative; FN, false negative; PPV, positive predictive value; NPV, negative predictive value.

As VS confirmation can only be for a day of ovulation in a positive cycle, there are no TN results; hence, only sensitivity and PPV results are available. The result can still be an FN for a comparator method (TOS or SWS) if it is unable to predict a positive result because the median value for the day of ovulation in previous cycles cannot be calculated due to anovulatory results, but VS is capable of predicting ovulation based on previous cycle results. VS and SWS data produce similar accuracy and F scores, with both accuracy measures dragged down for TOS by the number of FNs.

The ovulation prediction performance of Days Difference distributions in the Confirmed Prior Diagnosis and Confirmed No Diagnosis groups did not differ significantly for VS (*p* = 0.4534), TOS (*p* = 0.2778), or SWS (*p* = 0.5226); and therefore, it is valid to compare the Days Difference and Threshold results for TOS and SWS between the two groups.

VS and SWS methods produce reduced accuracy for the Confirmed Prior Diagnosis group, which bears out the higher standard days deviation results for that group presented in Table 12, whereas the TOS method produced very similar accuracy between the two groups.

The ovulation prediction performance of Days Difference distributions in the Late Ovulation and Confirmed Early + Normal Ovulation Timing groups differed significantly for VS (*p* = 0.006) and TOS (*p* = 0.0151) but did not differ significantly for SWS (*p* = 0.0817), and therefore, although it may be valid to compare the Days Difference and Threshold results for TOS and SWS between the two groups for SWS, caution should be exercised in doing so for VS and TOS.

The accuracy and F scores are very low in the Late Ovulation group, although VS and SWS produce exactly the same results. VS produces the highest accuracy for the Early + Normal Ovulation Timing group, but the accuracy scores for TOS are lower.

## 4 Discussion

### 4.1 Introduction

It should be remembered before discussing the results that by recording temperature intravaginally, VS is measuring a different temperature curve from SWS. The hypothesis for this study based on previous research (Papaioannou et al., 2013a; Papaioannou et al., 2013b; Papaioannou et al., 2014; Regidor et al., 2018) is that VS provides the most accurate representation of the thermogenic effect of progesterone released during ovulation and can hence be treated as a “gold standard” ovulation result for each side-by-side measured cycle for comparative purposes with the TOS and SWS methods.

In general, for all the group analyses, the high FN rate for TOS was largely the result of strict application of the method rules, with insufficient data points available to determine three high values in a window of six consecutive days. The higher FP rate for the SWS method vs. TOS for ±1 day but a lower rate for ±3 days shows the “spring-loaded beam” algorithm employed by SWS is probably better at adjusting for the overall bi-phasic pattern around the ovulation window than for producing exact agreement with VS.

For all group analyses, the Days Difference Method mean days difference results show that TOS and SWS confirmations of ovulation are earlier in the cycle than VS. TOS results are generally earlier than SWS, but the standard deviation of most analyses show a lower variability between the TOS and SWS results than the mean days difference would suggest alone.

### 4.2 Data Set Results

As would be expected, the Training Data Set produced higher SWS accuracy for both ±1 day and ±3 days analyses. SWS outperforms TOS by all measures in comparison to the VS results, but the TOS accuracy was very similar for Training and Additional data sets, which may indicate that the SWS algorithm was “over-tuned” to the Training Set. As can also be expected, the F score measure varied less than the accuracy measure between the three sets.

It should be noted that the Mann–Whitney test revealed a significant difference between the Training Set and Additional Set groups (*p* = 0.0291), and this might indicate a greater span of median cycle lengths and hence cycle variability in the Additional Data Set group. This may be responsible for the poorer performance of the algorithm with the Additional Data Set group, although the individual Mann–Whitney tests on ovulation confirmation would indicate no significant difference in performance between the Training Set and Additional Set groups using either the TOS (*p* = 0.1948) or SWS (*p* = 0.3132) methods.

### 4.3 Skin Site Results

As discussed in the *Introduction*, few papers have established the accuracy of skin-based temperature measurement for the determination of the date of ovulation, and to the authors' knowledge by means of a literature search conducted for this paper, none to date has provided a comparative assessment of the same device in differing skin sites for this purpose, although a number of papers have attempted to establish the best approach to skin temperature monitoring as detailed in the literature review by MacRae et al. (MacRae et al., 2018).

Intuitively, by offering a greater skin contact surface area that is more shielded from ambient temperature, the arm site should provide a more consistent temperature result from night to night and therefore a more accurate measure of ovulation than measuring on the underside of the wrist. However, in this analysis, the Wrist site outperformed the Arm for accuracy as measured by the accuracy and F score. This may be due to better contact due to the wristband vs. the armband design or that the Wrist participants complied more closely with the protocol for wearing the sensor on the wrist. MacRae et al. (2018) pointed out that the more insulated the temperature sensor is from the ambient temperature (as is the case for the Arm site of SWS vs. the Wrist site), the higher the resulting measured temperature is likely to be, but it is likely these conditions remain relatively constant from night to night for an individual user and hence do no significantly affect a system where relative changes in temperature over time rather than absolute temperature is the key to functionality.

However, the smaller sample size in the Wrist group may have nonetheless biased the results in a way that did not affect median cycle length or proportion of +ve and −ve results, as there are noticeably fewer SWS FP and FN results (6% of cycles) for the Wrist group compared with the Arm group (11% of cycles) for the ±3 days threshold. Likewise, the TOS FP and FN results (6% of cycles) for the Wrist group compared with the Arm Group (22% of cycles) explain the larger discrepancy in its accuracy. Nonetheless, the lack of significant difference between the groups as shown by the Mann–Whitney test combined with the similar results between the modalities is encouraging, as it indicates that SWS can be made to work equally well on either the Wrist or Arm, within the precision of this study. It follows that by applying similar sensor construction and algorithm techniques, other systems could also be made to work as well for the underside of the wrist as well as the arm. Some existing products use the top side of the wrist for temperature measurement (Goodale et al., 2019; Zhu et al., 2021), and the results from this study cannot therefore be used to draw an inference of potential accuracy for those systems.

### 4.4 Diagnosis and Ovulation Timing Results

A number of clinically useful observations can be drawn from the analysis of prior Diagnosis and Ovulation Timing.

1) *For this study population*, 28% of participants who confirmed a prior diagnosis associated with ovulatory dysfunction (17 out of 61 participants) recorded cycles that only showed Early + Normal Ovulation Timing. This is an unexpected result and indicates some of the challenges in trying to understand what represents a “normal” patient.
2) Late Ovulation is prevalent in this study population, and given 25% of cycles recorded by participants indicating Confirmed No Diagnosis showed Late Ovulation, it is possible that ovulatory dysfunction goes undetected. This reflects previous clinical findings (Lenton et al., 1984) and explains perhaps why the use of prior cycle results for the prediction of ovulation in subsequent cycles is generally problematic.
3) Although the usefulness of knowing the exact date of ovulation in a prior cycle can be questioned, it is generally accepted that better knowledge of the fertile window provides a higher chance of conception and that the more accurately this is known, the higher the chance of conception (Wilcox et al., 1995; Dunson et al., 2002).
4) With an accuracy of 90% and F score of 0.93 for the ±3 days threshold, SWS appears to be a more accurate method for the confirmation of the fertile window than previously studied methods of oral temperature (Freundl et al., 2003) (accuracy 78%, F score 0.88) and skin temperature (Goodale et al., 2019; Zhu et al., 2021) (F score of 0.78) in determining the fertile window. However, comparison of these results with other studies should be treated with caution, as it is carried out on a distinct user population with a distinct device and algorithm.
5) The detection of the absence of ovulation is perhaps one of the most important potential uses for temperature in monitoring fertility, but anovulatory cycles are often excluded from analysis in studies, as they reduce the amount of usable data for determining the date of ovulation. The study presented 47 anovulatory cycles in a total of 205 cycles. This significant number of anovulatory cycles provides a much better understanding of true accuracy, and SWS would appear to be capable of detecting anovulation when compared to VS results and by-eye cycle assessment.
6) There appears to be slightly lower accuracy in the confirmation of ovulation by SWS in the Confirmed Prior Diagnosis group (±3 days accuracy 89%, F score 0.92) than those with Confirmed No Diagnosis (accuracy 93%, F score 0.95). Although the groups were found to differ significantly in median cycle length (p = 0.0131), which may account for the difference, the ovulation confirmation performance of the methods themselves TOS (p = 0.6518) and SWS (p = 0.3182) did not differ significantly between the two groups, so the difference in the accuracy and F score is more likely to be a result of the performance of the methods in respect of negative results.
7) There appears to be a smaller difference in accuracy in the confirmation of ovulation by SWS for those with Late Ovulation (±3 days accuracy 87%, F score 0.93) vs. Early + Normal Ovulation Timing (accuracy 90%, F score 0.94), although again the groups differed significantly (p < 0.001). This would indicate SWS can be used effectively in a population with ovulatory dysfunction whether it is previously known or not. This is also a useful result given the drawbacks of BBT outlined by previous clinical literature (Lenton et al., 1977; Martinez et al., 1992; Ayres-de-Campos et al., 1995; Barron and Fehring, 2005; Mazerolle et al., 2011).

### 4.5 Prediction Results

With a ±3 days threshold accuracy result of 60.0% (CI 48.8%–70.5%), SWS provides a surprisingly similar prediction result to VS, which produces a ±3 days threshold prediction accuracy of 63.5% (CI 52.4%–73.7%) compared with its own confirmatory data for the next cycle. The F scores are similarly close: SWS 0.75 and VS 0.78.

The ±3 days prediction accuracy is improved for both methods: SWS = 66.7% (CI 53.3%–78.3%) and VS = 71.7% (CI 58.6%–82.5%) when Late Ovulation results are removed, so the identification of such cycles would appear to be helpful for women with ovulatory dysfunction when trying to conceive naturally. This conclusion may also lead to future algorithm improvements.

Nevertheless, real-time in-cycle prediction using current cycle data remains important for aiding conception and offers significant benefits over predictions based on prior cycle results. While urinary LH provides these in-cycle results, clinical publications have highlighted the inaccuracy of LH, particularly for those with ovulatory dysfunction (Lloyd and Coulam, 1989; Robinson et al., 1992; McGovern et al., 2004). The VS in-cycle method was developed to overcome this issue and has been reported upon previously (Papaioannou et al., 2014).

## 5 Conclusion

Although ovulatory dysfunction and luteal phase abnormalities may be causes of infertility or early pregnancy loss, clinical assessment of the luteal phase has proven challenging (Practice Committees of the American Society for Reproductive Medicine and the Society for Reproductive Endocrinology and Infertility, 2021). Luteal progesterone production is stimulated by LH pulses. The variability of serum progesterone levels from day to day and hour to hour limits the utility of measuring a single progesterone level to assess the overall adequacy of progesterone production during the luteal phase. Assessing multiple progesterone levels over several days and determining the area under the curve has been used to demonstrate luteal phase abnormalities, but this type of evaluation is not feasible in a clinical setting. Fertility may be impaired in women who experience a short luteal phase, but since urine LH tests are unreliable for some women, misinterpretation of the data or misdiagnosis may occur. The endometrium undergoes histologic and molecular changes throughout the postovulatory interval, but the reliability and reproducibility of endometrial biopsy have been questioned. To date, there is no universally accepted method to adequately assess the luteal phase.

Assessing the thermogenic effect of progesterone on core body temperature by VS or SWS provides a simple and reproducible method to confirm ovulation and assess the luteal phase. In this study, we have shown that VS and SWS are useful tools for confirming the fertile window and assessing for ovulation and anovulation for women's ovulatory dysfunction.

### 5.1 Contribution to the Field Statement

The number of female fertility monitoring tools available on the market has increased markedly over the past 5 years, mainly due to the advent of mobile devices, facilitating the development of personal period and fertility tracking applications. However, without accurate sensors and analysis algorithms, these have no way of confirming the presence or absence of ovulation. Where applications do use sensors, accuracy for predicting ovulation in a subsequent cycle has been generally poorly examined in the literature, in particular with respect to comparator methods. Indeed, a number of publications question using past cycles for predicting ovulation in subsequent cycles (Setton et al., 2016), given estimates of only approximately 21% accuracy (Johnson et al., 2018).

This paper examines a novel approach to confirmation of ovulation and prediction for subsequent cycles when compared to an established independent comparator method. It was of further interest to determine its value in a population with prior diagnoses known to cause ovulatory dysfunction, as well as its ability to diagnose possible ovulatory dysfunction in users with no prior diagnosis.

The conclusions are that temperature-based sensors increase the accuracy of ovulation determination over the use of historic data alone and therefore significantly benefit those trying to conceive naturally. Furthermore, a more convenient skin-worn sensor, with appropriate data analysis, can be almost as useful as a vaginal sensor for real-time ovulation detection and as such is especially useful for women with irregular cycles.

Both kinds of sensors can also contribute to the screening for ovulatory dysfunction, even when it is less evident because cycle lengths appear to be in the expected range and as such better inform women struggling to conceive by helping them understand their cycles, reducing unnecessary uncertainty and stress, and providing practical advice on when to attempt conception.

### 5.2 Implications for Further Research

The “spring-loaded beam” temperature analysis method appears to provide a more accurate result than the “TOS” (Barrett and Marshall, 1969; McCarthy and Rockette, 1983) rule for determining when ovulation has taken place. It also is better at removing anomalous results produced by “TOS” where there is a clear bi-phasic temperature shift (by manual plotting and then by eye), but the hard mathematical rule cannot be applied as a result of too few data points, or data points lying outside of the “low” or “high” sets.

Furthermore, the “spring-loaded beam” method would be expected to have similar advantages for data obtained by other temperature measurements and hence improve the accuracy of results obtained by other temperature measurements, particularly oral temperature.

The SWS algorithm should now be reassessed in light of the Additional Set results.

It is unclear whether the median methodology for Prediction from prior cycle results is beneficial or in fact a hindrance to accuracy compared with a simpler method of (for instance) just using the most recent prior cycle result. The extension of the multiple serial cycles data set should assist with the creation and testing of further algorithmic predictive techniques.

Given the relative similarity in ovulation confirmation results between SWS and VS, using current cycle data to predict the onset of ovulation using an adapted algorithm might be possible with SWS. Adding to the current data set, in particular adding serial cycles with “normal” ovulation timing from women without known ovulatory dysfunction, would aid such a development. Equally, understanding whether a user of such a device ovulates late in their cycle and adjusting the algorithm to intelligently exclude or use those cycles through machine learning techniques could enhance the accuracy of prediction.

Additional confirmatory methods, in particular multiple LH and ultrasound follicular monitoring results, would greatly assist in establishing accuracy for a broader user population and demographic, of both Confirmation of the day of ovulation and Prediction of the ovulation day for a subsequent cycle and in the development of in-cycle prediction.

## Data Availability

The original contributions presented in the study are included in the article/Supplementary Material, further inquiries can be directed to the corresponding author.
